# Prediction of peak oxygen consumption using cardiorespiratory parameters from warmup and submaximal stage of treadmill cardiopulmonary exercise test

**DOI:** 10.1371/journal.pone.0291706

**Published:** 2024-01-10

**Authors:** Maciej Rosoł, Monika Petelczyc, Jakub S. Gąsior, Marcel Młyńczak

**Affiliations:** 1 Faculty of Mechatronics, Institute of Metrology and Biomedical Engineering, Warsaw University of Technology, Warsaw, Poland; 2 Faculty of Physics, Warsaw University of Technology, Warsaw, Poland; 3 Department of Pediatric Cardiology and General Pediatrics, Medical University of Warsaw, Warsaw, Poland; Instituto Politecnico de Setubal, PORTUGAL

## Abstract

This study investigates the quality of peak oxygen consumption (VO_2peak_) prediction based on cardiac and respiratory parameters calculated from warmup and submaximal stages of treadmill cardiopulmonary exercise test (CPET) using machine learning (ML) techniques and assesses the importance of respiratory parameters for the prediction outcome. The database consists of the following parameters: heart rate (HR), respiratory rate (RespRate), pulmonary ventilation (VE), oxygen consumption (VO_2_) and carbon dioxide production (VCO_2_) obtained from 369 treadmill CPETs. Combinations of features calculated based on the HR, VE and RespRate time-series from different stages of CPET were used to create 11 datasets for VO_2peak_ prediction. Thirteen ML algorithms were employed, and model performances were evaluated using cross-validation with mean absolute percentage error (MAPE), R^2^ score, mean absolute error (MAE), and root mean squared error (RMSE) calculated after each iteration of the validation. The results demonstrated that incorporating respiratory-based features improves the prediction of VO_2peak_. The best results in terms of R^2^ score (0.47) and RMSE (5.78) were obtained for the dataset which included both cardiac- and respiratory-based features from CPET up to 85% of age-predicted HR_max_, while the best results in terms of MAPE (10.5%) and MAE (4.63) were obtained for the dataset containing cardiorespiratory features from the last 30 seconds of warmup. The study showed the potential of using ML models based on cardiorespiratory features from submaximal tests for prediction of VO_2peak_ and highlights the importance of the monitoring of respiratory signals, enabling to include respiratory parameters into the analysis. Presented approach offers a feasible alternative to direct VO_2peak_ measurement, especially when specialized equipment is limited or unavailable.

## 1. Introduction

Peak oxygen consumption (VO_2peak_) obtained through cardiopulmonary exercise test (CPET) is the popular measure of cardiorespiratory fitness [1]. It is a reliable predictor of cardiac events [2, 3], as well as lung cancer [4] and liver transplantation survival [5] and risk of postoperative complications [6]. Moreover, VO_2peak_ is a predictor of sport performance [7–9] and physical task performance during spaceflight [10]. Although CPET is the most reliable form of test, it is costly, requires specialized personnel and advanced equipment [11].

While conducting CPET, heart rate (HR) data are usually obtained through electrocardiography (ECG), while respiratory rate (RespRate) and pulmonary ventilation (VE) are gathered using tight-fitting masks. Nevertheless, this data can be acquired with relative ease, using heart rate monitors or smartwatches in case of HR, and impedance pneumography (IP) in case of RespRate and VE [12, 13]. Moreover, CPET is physically demanding as assumes the participants’ exhaustion and thus it is contraindicated for patients with acute myocardial infarction, unstable angina, uncontrolled arrhythmia causing symptoms or hemodynamic compromise, uncontrolled asthma, and other pathological conditions [11]. Maximal cardiopulmonary exercise test might also interfere with an athletes training program [14].

Actually, due to: a) the growing development of aforementioned measurement devices, b) availability of simply field-based tests such as incremental shuttle walk test [15, 16] and c) new statistical prediction models and equations, clinicians and/or researchers are able to estimate VO_2peak_, and/or VO_2max_ (it is however not the subject of this study), based on selected parameters without performing maximal CPET [17–22]. Unfortunately, estimated VO_2peak_ using, e.g., only 6-min Walk Test distance demonstrated poor agreement with measured VO_2peak_ from a CPET [23]. Addition of other data such as demographic, anthropometric, and functional characteristics improved the accuracy of VO_2peak_ estimate based on walking tests at least in elderly patients with stable coronary artery disease (model with all variables explained 73% of VO_2peak_ variance) [24]. Thus, estimation of peak oxygen consumption based on combination of demographic factors and cardiac parameters obtained during submaximal (or even not) physical effort is possible, however, may be biased.

Reliable and accurate estimation of VO_2peak_ without performing maximal CPET may require more input physiological data to perform more sophisticated analyses. Thus, the development of new prediction models or equations, which will be able to accurately estimate VO_2peak_, and/or VO_2max_, and will not relies on performing maximal CPET, is still ongoing [18, 25]. In recent years with the growth of the popularity of ML tools incorporated during the data analysis phase, those techniques were also utilized for the prediction of VO_2_ kinetics and VO_2max_ [26, 27]. ML models were also used by *Szijarto et al*. for prediction of VO_2peak_ based on the anthropometric data and 2D echocardiography (2DE) [28]. This approach was more accurate than a model based on anthropometric factors, however, it required performing a 2DE examination with sophisticated equipment and a trained physician. Importantly, not only the model or prediction algorithm might be important in terms of the prediction accuracy, but also the features used for the training. There are existing studies utilizing respiratory rate and ML for prediction of oxygen uptake dynamics during CPET [29–31]. However, to the best of our knowledge, there have been no previous studies utilizing features from cardiorespiratory time-series obtained from submaximal CPET, for the prediction of VO_2peak_ using ML models.

The aim of this paper was hence to investigate the quality of VO_2peak_ prediction by models based on cardiac and respiratory features obtained from different stages of CPET. Additionally, we assessed the importance of respiratory-based features included in the models for VO_2peak_ prediction.

## 2. Materials and methods

### 2.1. Data and study population

The study was performed on the publicly available database of cardiorespiratory time-series acquired during treadmill maximal cardiopulmonary exercise tests presented by *Mongin et al*. [32, 33]. The database comprises 992 recordings from experiments undertaken among amateur and professional athletes in the Exercise Physiology and Human Performance Lab of the University of Málaga between 2008 and 2018 with two types of protocols: a continuous increase of treadmill speed and a graded approach. In the database, one may find two forms of protocols on the treadmill continuous (ramping) and step-by-step incremental effort [32, 33]. Discussing the consistency of the analysis, we decided to limit our study only to experiments with continuous increase of the speed. In general, the protocol has: warmup lasting 8–10 minutes at 5km/h (recording covers only about two last minutes), incremental effort with a 1km/h/min speed increase and recovery. In the latter phase, the treadmill speed was set back to the initial 5 km/h speed [33]. The length of recordings from warmup differs between cases but was satisfactory for our modeling purposes. During recovery, subjects were asked to walk. Participants were instructed to go beyond exhaustion and the test was considered maximal if the increase of VO_2_ was less than 2.1 mL/kg/min between successive stages. Then the effort test was stopped “to avoid the vasovagal syncope” [32, 33] and the recovery started. The study was conducted according to the principles of the Declaration of Helsinki, the study protocol was approved by the Research Ethics Committee of the University of Málaga, written informed consent was obtained from the participants and all the data were analyzed anonymously.

During each test, the following cardiorespiratory time-series were acquired: heart rate (HR), respiratory rate (RespRate), pulmonary ventilation (VE), oxygen uptake (VO_2_) and carbon dioxide production (VCO_2_). Data were acquired on a breath-to-breath basis. HR was monitored via a 12-lead ECG (Mortara Instrument, Inc., USA), while respiratory signals were obtained using the CPX MedGraphics gas analyzer system (Medical Graphics Corporation, USA) [32].

Participants between 18 and 40 years old were chosen for the analysis reducing the sample size to 692. Tests only with continuous increasing speed were selected in order to obtain more consistent conditions along the study population. In result, 485 recordings have left. Next, subjects who were determined as outliers based on the 1.5 interquartile range method in terms of weight, height, and VO_2peak_, with respect to the given sex, were excluded from the study, limiting to 462 recordings. Furthermore, the obtained data was visually evaluated in order to discard measurements during which there were visible artefacts in HR acquisition (e.g., sudden drop of over 30 bpm or lack of continuity of HR time-series during CPET probably due to electrode detachment); ultimately 369 recordings became background for the analysis. The final recordings belong to 327 unique subjects (42 subjects had more than one test) including 275 men and 52 women. The demographic summary of the final group is presented in Table 1.

**Table 1:** Descriptive statistics of the study population.

	Age [years]	Height [cm]	Weight [kg]	BMI	VO_2peak_ [ml/min/kg]
Men	27.3 ± 5.8 (18.0–39.8)	177.4 ± 6.3 (160.5–193.0)	76.6 ± 8.3 (55.3–97.0)	24.3 ± 2.2 (17.9–31.4)	47.7 ± 7.5 (28.9–67.3)
Woman	26.9 ± 6.3 (18.0–40.0)	165.2 ± 6.1 (154.0–178.0)	62.2 ± 8.2 (46.0–83.0)	22.8 ± 2.3 (18.0–29.6)	38.1 ± 6.3 (24.8–53.8)
All	27.3 ± 5.9 (18–40)	175.5 ± 7.6 (154.0–193.0)	74.5 ± 9.7 (46.0–97.0)	24.1 ± 2.3 (17.9–31.3)	46.3 ± 8.1 (24.8–67.3)

### 2.2. Modeling

Based on the aforementioned dataset, we decided to investigate the quality of VO_2peak_ prediction from different stages of CPET based on cardiac and respiratory parameters, and to assess the importance of respiratory-based features included in the modeling of VO_2peak_. For this purpose, we utilized recorded time-series of HR, RespRate, and VE. VO_2peak_ was determined as the maximal value of the signal obtained after a 15-breath VO_2_ moving average window according to the recommendation presented by *Robergs et al*. [34].

**Fig 1:**
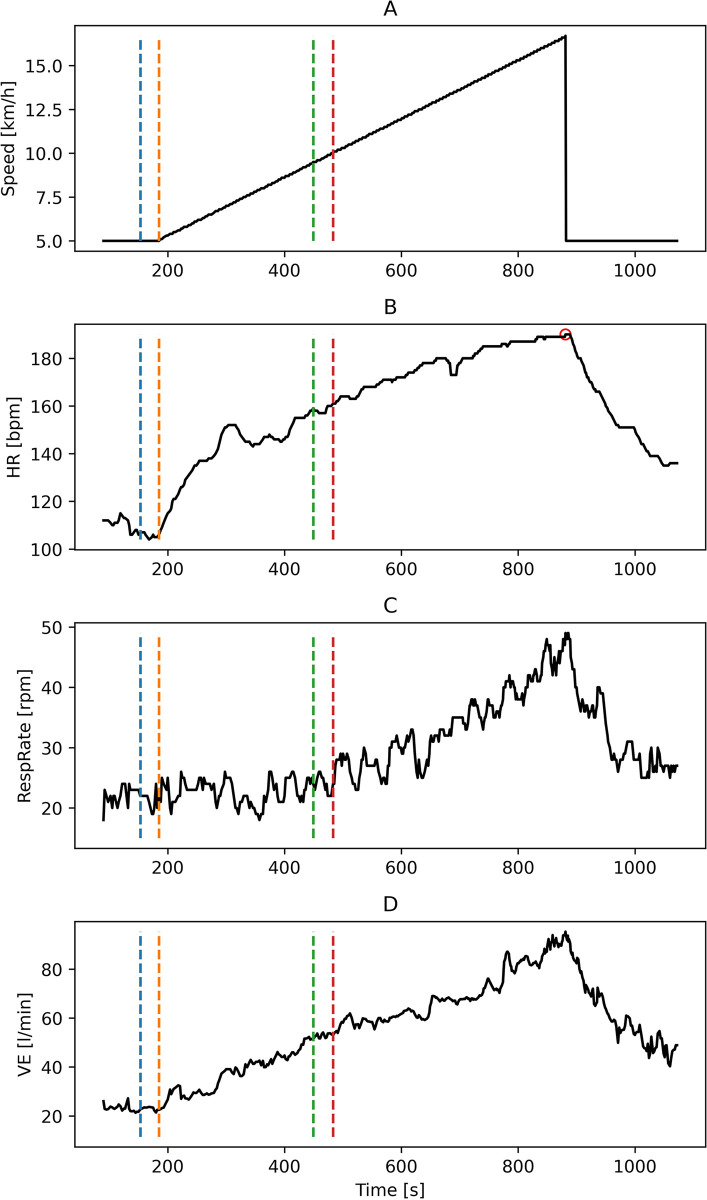
Typical representation of the time-series for participants with selected fragments used in the analysis. Part A presents the linearly increasing treadmill speed, part B heart rate fluctuations, part C respiratory rate and part D pulmonary ventilation kinetics. The segment between the blue and orange dashed lines is the last 30 seconds of warmup. The segment between the orange and green lines corresponds to the section of CPET up to 85% of the age-predicted HR_max_. Finally, the segment between the orange and red lines corresponds to the increasing workload in CPET up to 85% of the measured HR_max_, which is marked with a red circle on the HR plot.

As features for ML models, basic statistics such as mean, standard deviation, maximal and minimal value, median, 25^th^ and 75^th^ quantile, skewness, kurtosis, coefficient from linear regression, impulse and shape factors were calculated for HR, RespRate, and VE, for a given stage of the maximal CPET. On this basis, 11 datasets were created based on different combinations of parameters and CPET stages, as presented in Table 2. Our research is focused on the submaximal stage from the cardiopulmonary exercise test, which equals 85% of the maximal measured and age-predicted HR_max_ as a threshold. Studied value of HR termination is commonly used in submaximal testing [35–37]. We also used both actual HR_max_ obtained during the treadmill cardiopulmonary exercise test, and age-predicted HR_max_ (220-age) in order to provide insights about the utility of the prediction of VO_2peak_ in submaximal tests without prior knowledge about the value of HR_max_ for a given subject. The example plot of the signals, alongside the threshold for all the stages of the CPET for which the features were calculated, is presented in Fig 1.

**Table 2:** Characteristics of all datasets with an indication of features belonging to individual datasets.

Dataset	Subjects’ demography (age, weight, height, sex)	HR features from the last 30 s of warmup	RespRate and VE features from the last 30 s of warmup	HR features from CPET up to 85% of HR_max_	RespRate and VE features from CPET up to 85% of HR_max_	HR features from CPET up to 85% of age-predicted HR_max_	RespRate and VE features from CPET up to 85% of age-predicted HR_max_
D1	+						
D2	+	+					
D3	+	+	+				
D4	+			+			
D5	+			+	+		
D6	+	+		+			
D7	+	+	+	+	+		
D8	+					+	
D9	+					+	+
D10	+	+				+	
D11	+	+	+			+	+

The 10-fold cross-validation (CV) was used to assess the accuracy of the prediction. In each iteration, standardization of the non-categorical features based on the mean and standard deviation from the training dataset was performed. The only feature that was not standardized was participants’ sex: -1 was assigned to male, and 1 to female subjects. Different ML algorithms, commonly used for regression problems, were utilized: Linear, Lasso and Ridge Regression, Random Forest, XGBoost, Multilayer perceptron, Epsilon-Support Vector Regression, Bayesian Ridge Regression, Bayesian Automatic Relevance Determination (ARD) Regression, Gaussian Process Regression, Gradient Boosting for Regression, Huber Regression and Theil-Sen Estimator [38–40]. The hyperparameter tuning was performed for each algorithm using the grid-search technique. In each iteration of the validation, metrics like mean absolute percentage error (MAPE), R^2^ score, mean absolute error (MAE), root mean squared error (RMSE) and Cohen’s f^2^ for effect size were calculated. The best model for each dataset was determined based one the lowest MAPE score (which was chosen arbitrarily) obtained from the cross-validation. For the best model, Lin concordance correlation coefficient was calculated, and results were visualized as the dependency between predicted and actual values of VO_2peak_ and as Bland-Altman plot. Moreover, the difference in the values of metrics for male and female was tested.

**Fig 2:**
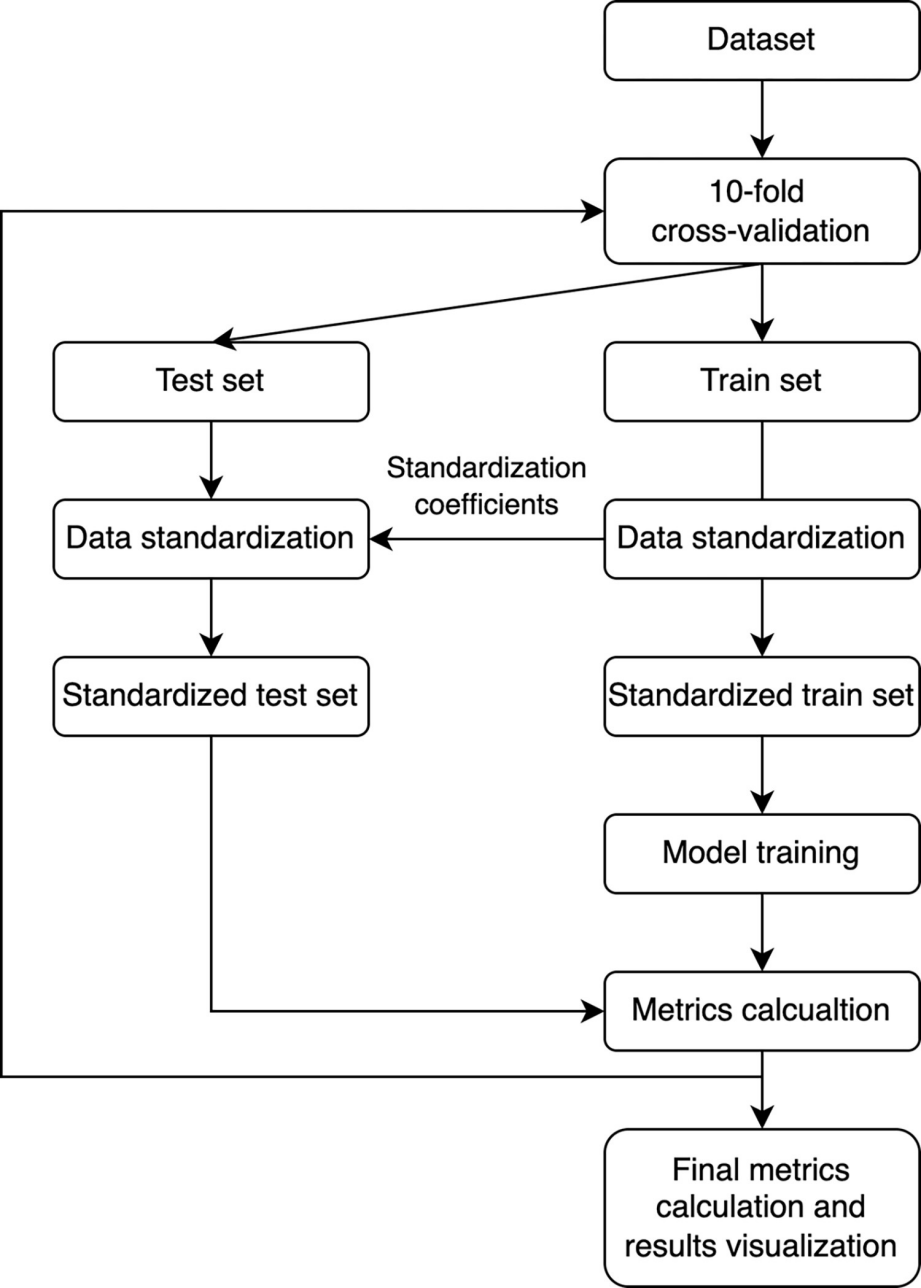
Modeling pipeline applied for each dataset and algorithm.

Metrics obtained from all datasets were pairwise compared using the Wilcoxon signed-rank test. The significance level was set to 0.05. For the calculations, Python 3.9.13 was used. The whole modeling pipeline is presented in Fig 2.

### 2.3. Explainable AI

In order to investigate the importance of the individual features used for ML modeling, explainable artificial intelligence (XAI) tools were applied. For this purpose, the Dalex Python package was used [41]. During each iteration of the cross-validation, Shapley values and model-level variable importance based on drop-out loss values were calculated on the test set. After the whole cross-validation, all Shapley values for each sample and feature, as well as mean variable importance values were visualized. For the variable importance, *model*_*parts* function of *dalex*.*Explainer* class was used. 30 permutation rounds were performed on each variable with MAE as a loss function and no data sampling (argument *N* was equal to *None*) due to the small number of samples.

## 3. Results

**Fig 3:**
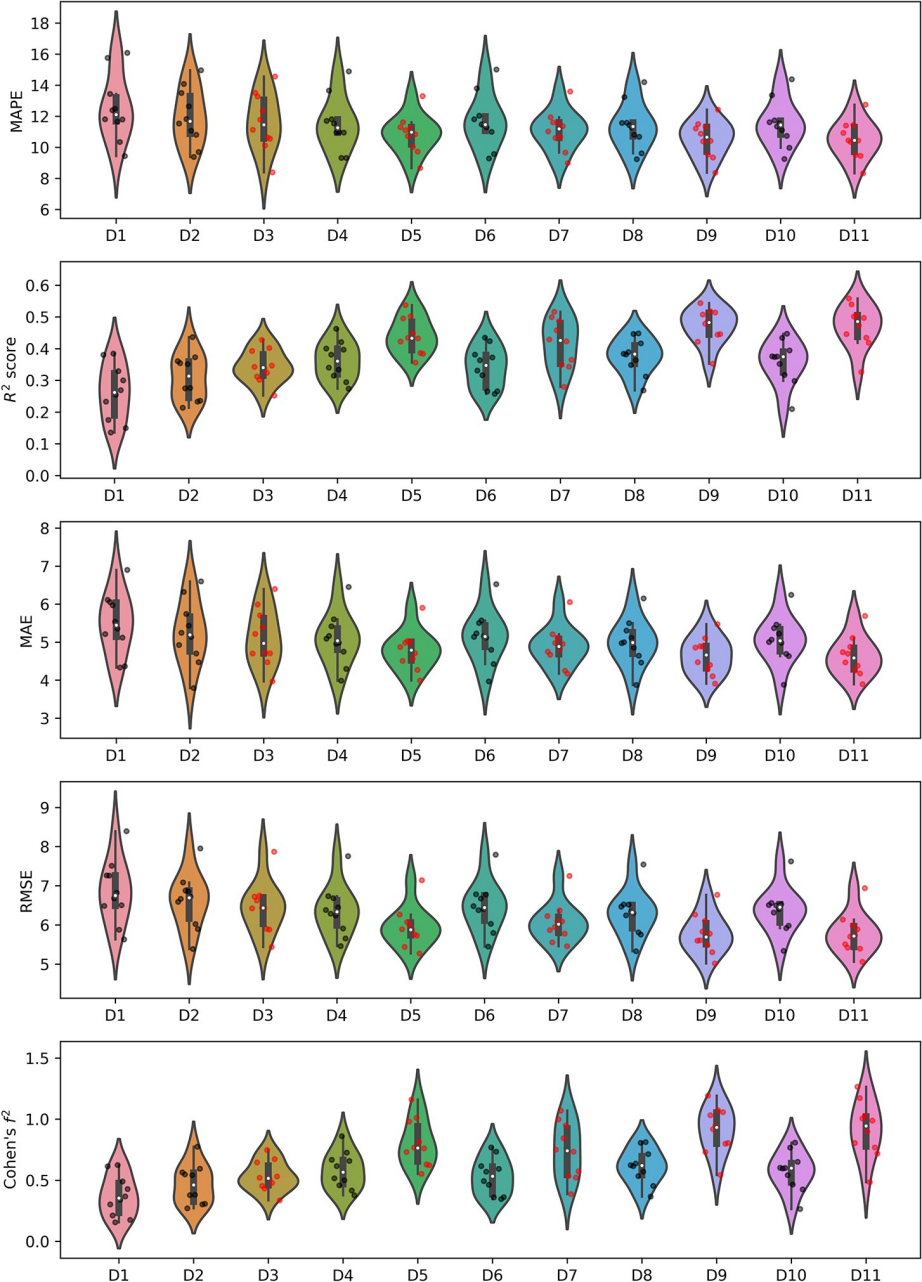
Violin-plots of the calculated metrics for each dataset with the visualization of the metrics obtained in each iteration of the 10-fold cross-validation. Black dots represent metrics obtained from datasets without respiratory-based features, while red dots represent these that include such features.

**Fig 4:**
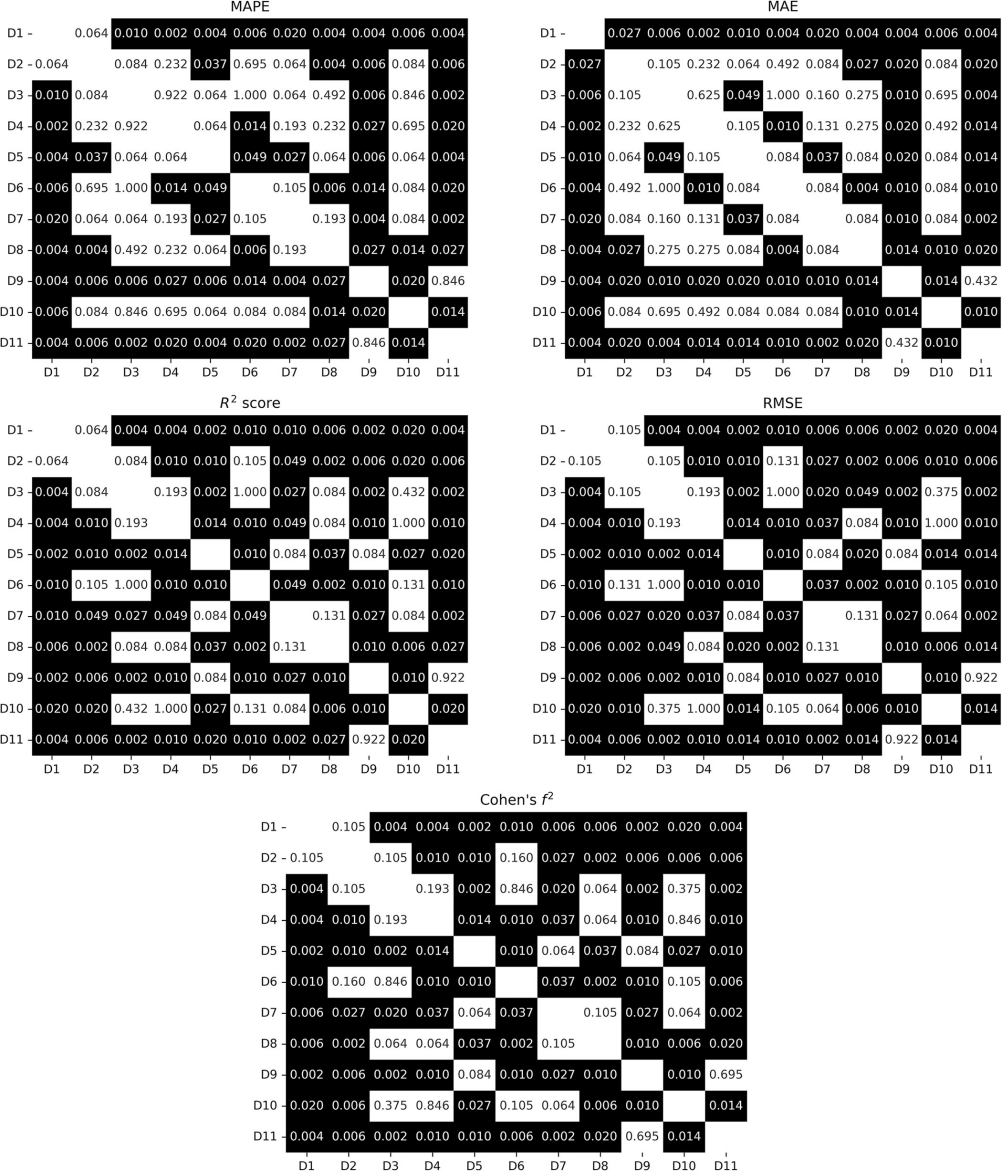
The p-values from Wilcoxon signed-rank test from pairwise comparison of the metrics obtained from different datasets. P-values smaller than 0.05 are marked with a black background.

The metrics obtained for the best algorithm in terms of the lowest MAPE from the cross-validation for each dataset are presented in Table 3 alongside the model names. The violin-plots of the obtained metrics for each dataset were visualized in Fig 3. The p-values from the Wilcoxon signed-rank test from a pairwise comparison of the metrics are presented in Fig 4.

**Table 3:** Mean and standard deviation of metrics from cross-validation for each dataset for the model which resulted in the lowest MAPE for the given dataset. The highest metric values were highlighted.

	MAPE [%]	R^2^	MAE [ml/min/kg]	RMSE [ml/min/kg]	Cohen’s f^2^	Model
D1	12.52 ± 2.11	0.26 ± 0.09	5.50 ± 0.80	6.84 ± 0.81	0.37 ± 0.17	Ridge regression
D2	11.95 ± 1.84	0.31 ± 0.07	5.24 ± 0.84	6.61 ± 0.71	0.47 ± 0.16	Huber regression
D3	11.63 ± 1.84	0.34 ± 0.05	5.13 ± 0.75	6.45 ± 0.65	0.53 ± 0.12	Bayesian ARD regression
D4	11.51 ± 1.72	0.36 ± 0.06	5.07 ± 0.68	6.36 ± 0.64	0.58 ± 0.15	Bayesian ARD regression
D5	10.86 ± 1.23	0.44 ± 0.06	4.78 ± 0.52	5.95 ± 0.51	0.80 ± 0.19	Bayesian ARD regression
D6	11.67 ± 1.72	0.34 ± 0.07	5.15 ± 0.69	6.46 ± 0.64	0.53 ± 0.15	Lasso regression
D7	11.10 ± 1.26	0.42 ± 0.08	4.90 ± 0.53	6.07 ± 0.50	0.74 ± 0.23	Bayesian ARD regression
D8	11.36 ± 1.49	0.38 ± 0.06	4.99 ± 0.61	6.29 ± 0.59	0.61 ± 0.14	Bayesian ARD regression
D9	10.54 ± 1.20	**0.47 ± 0.06**	4.64 ± 0.49	**5.78 ± 0.50**	0.91 ± 0.19	Bayesian ARD regression
D10	11.50 ± 1.49	0.36 ± 0.07	5.06 ± 0.62	6.37 ± 0.59	0.57 ± 0.16	Bayesian ARD regression
D11	**10.51 ± 1.24**	0.47 ± 0.07	**4.63 ± 0.52**	5.78 ± 0.52	**0.91 ± 0.23**	Bayesian ARD regression

The lowest MAPE and MAE (10.51% and 4.63, respectively) were obtained for dataset D11 (demographic data along with cardiac and respiratory features from the last 30 seconds of warmup and CPET up to 85% of age-predicted HR_max_), while the lowest RMSE and highest R^2^ score (5.78 and 0.47, respectively) were obtained for D9 (demographic data along with cardiac and respiratory features from CPET up to 85% of age-predicted HR_max_). The worst prediction of VO_2peak_ in terms of all metrics was achieved by using the D1 (demographic data) dataset. Results obtained for D11 were statistically significantly better in terms of all metrics than results for all the rest of the datasets excluding D9 as presented in Fig 4. Regarding R^2^ score and RMSE metrics, datasets that included respiratory-based features from the part of CPET (irrespective of HR_max_ determination, whether measured or estimated) showed statistically significant superiority over datasets lacking features based on VE and respiratory rate during the corresponding period. Similarly, for MAPE and MAE, datasets containing respiratory-based features calculated up to 85% of age-predicted HR_max_ demonstrated significantly better metrics than datasets without such features. The effect size was large (Cohen’s f^2^>0.35) [42] for all iterations of cross-validations in the case D4, D5, D7, D8, D9 and D11.

**Fig 5:**
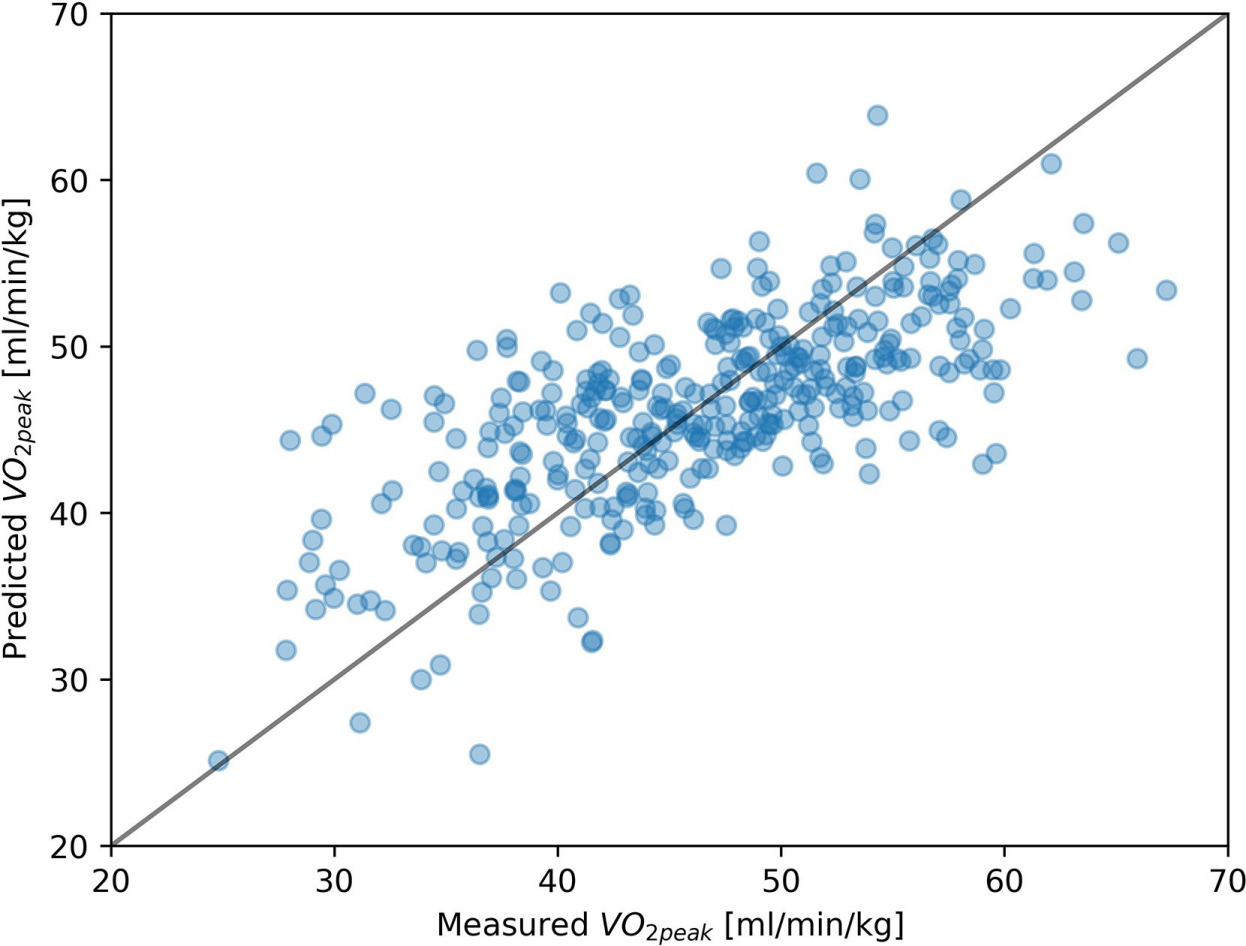
The plot of measured and predicted VO_2peak_ values for dataset D11. The solid black line represents the function where predicted value is equal to the measured one.

**Fig 6:**
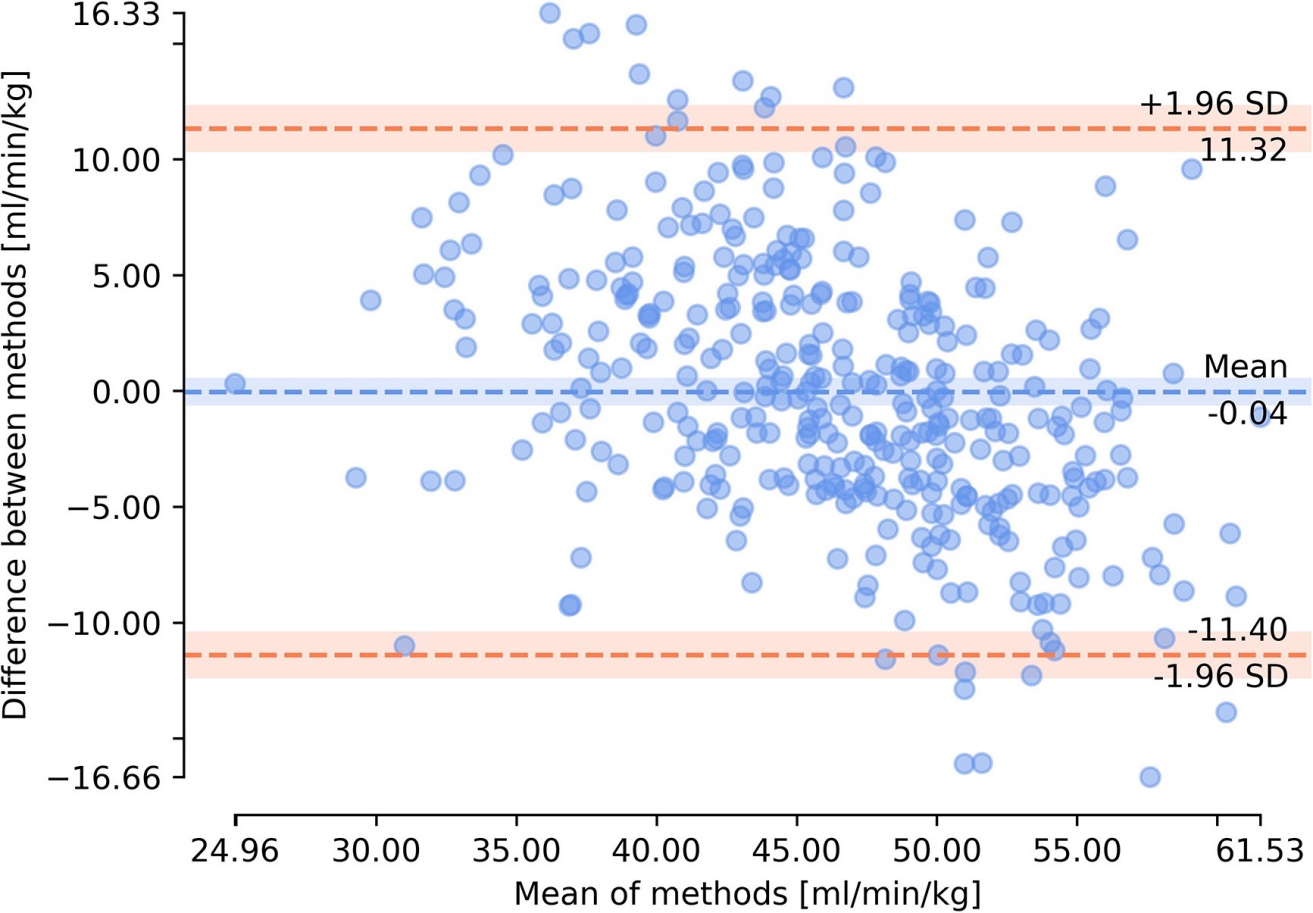
Bland-Altman plot of determined in CPET directly and predicted VO_2peak_ values based on the results for dataset D11.

The measured values of VO_2peak_ and values predicted for the dataset that obtained the lowest MAPE score (D11) were visualized in Fig 5. The Lin concordance correlation coefficient between predicted and measured VO_2peak_ values was 0.66. The Bland-Altman plot for this dataset is presented in Fig 6. There was no statistically significant difference in case of metrics obtained for male and female subjects. The results of the comparison of metrics obtained for male and female are presented in Table 4.

**Table 4:** Mean and standard deviation of metrics from cross-validation for male and female for dataset D11 with p-value from Wilcoxon signed-rank test.

	MAPE	R2	MAE	RMSE	Cohens f2
Male	10.29 ± 1.30	0.35 ± 0.12	4.69 ± 0.58	5.86 ± 0.51	0.58 ± 0.29
Female	11.77 ± 2.03	-0.16 ± 1.24	4.25 ± 0.70	5.16 ± 1.13	0.43 ± 0.76
P-value	0.105	0.232	0.160	0.105	0.557

**Fig 7:**
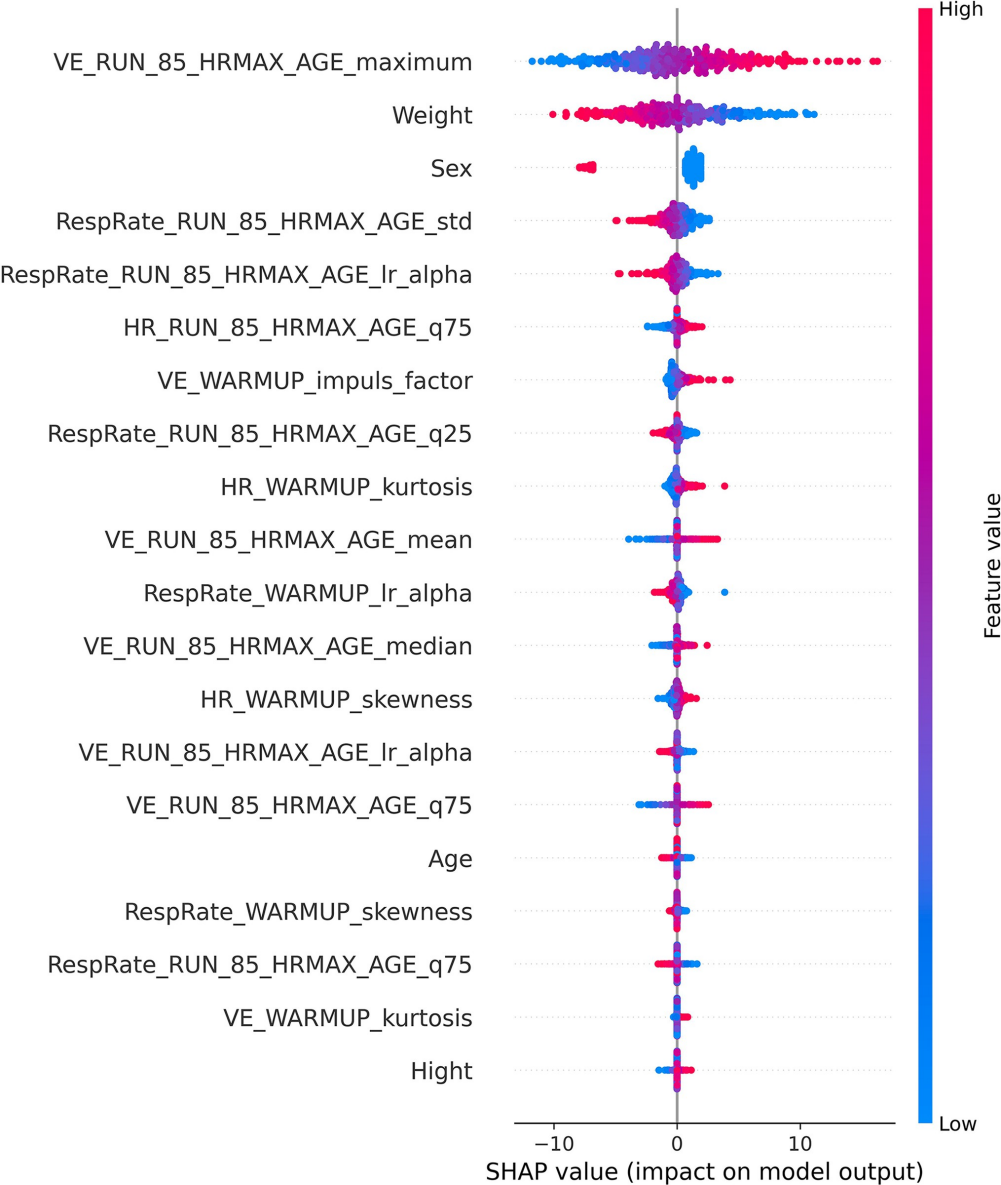
Shapley values obtained for dataset D11. Feature names are explained in the S1 Appendix.

**Fig 8:**
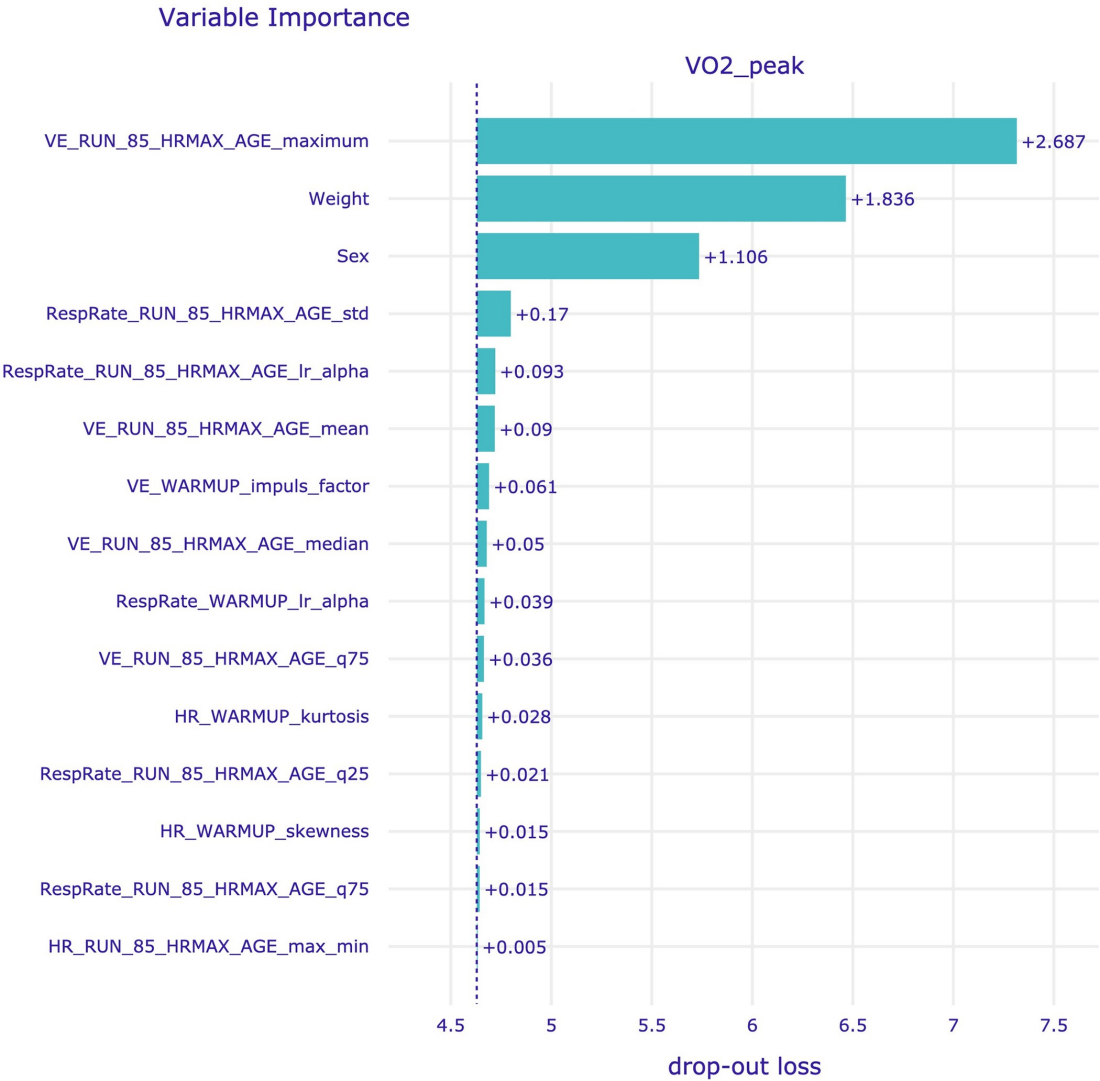
Variable importance for dataset D11. Feature names are explained in the S1 Appendix.

As the smallest mean MAPE was obtained for D11, Shapley values and feature importance were visualized for this dataset in Figs 7 and 8, respectively. The discussion of the XAI results can be found in the next section.

## 4. Discussion

Considering the features calculated from HR, VE, and RespRate time-series (attainable without the specialized equipment used in CPET), it is possible to predict VO_2peak_ from a submaximal test relying on age-predicted HR_max_, achieving a mean absolute percentage error of 10.51% (for D11), using Bayesian ARD regression method. The addition of respiratory-based parameters resulted in an improvement of prediction compared to datasets based solely on the corresponding stage of the treadmill cardiopulmonary exercise test in 4 out of 5 cases in terms of R^2^ score and RMSE, and 2 out of 5 cases in terms of MAPE and MAE. When limiting treadmill cardiopulmonary exercise test to 85% of age-based HR_max_, the inclusion of features based on VE and RespRate improved the prediction in terms of all the specified metrics. The fact that the best results were achieved for the dataset considering 85% aged-based HR_max_ and parameters obtained from easily accessible time-series indicates the possibility of using the presented method in clinical practice to determine VO_2peak_ without the prior knowledge of the actual HR_max_ value and the necessity to perform a maximal treadmill cardiopulmonary exercise test.

Obtaining VO_2peak_ from maximal CPET might be costly, time-consuming and in some cases impossible or contraindicated to carried out due to observed cardiac or pulmonary dysfunction, musculoskeletal diseases, or strict training programs. Therefore, there is a growing interest in the prediction of VO_2peak_ and/or VO_2max_ from submaximal tests [14, 43–49]. Our study focused on investigating ML algorithms to predict VO_2peak_ with the set of features, which could be obtained using simpler techniques than commonly used spirometry, and the significance of incorporating respiration into the prediction process. The presented results are similar or superior compared to some other presented VO_2peak_ prediction methods like WFI VO_2peak_ prediction equation, deep-learning model based on 2DE, or regression models from PACER 20-m shuttle run [19, 28, 50–53]. However, in the existing literature, there are also techniques, which managed to obtain better performance like regression models based on submaximal exercise test protocol using a total body recumbent stepper [54–56]. Nonetheless, in those studies more heterogeneous groups of patients were present in terms of age or health status (patients after heart failure or individuals with low to moderate risk of cardiovascular diseases). Further improvement of the prediction of VO_2peak_ might be achieved by increasing sample size, and inclusion of other parameters based on the raw signals (especially ECG) like HRV and parameters from information and causal domain [57–60].

Another notable aspect of the study was the utilization of XAI tools, specifically Shapley values and model-level variable importance, to obtain insights into the feature importance for prediction. For most datasets (including D9 and D11, which produced the best results) Bayesian ARD Regression model was used, which has an ability to automatically determine the relevance of each feature, effectively pruning irrelevant or redundant information, while accentuating the impactful variables [61]. In our analysis, we found that the top five most influential features were consistent between Shapley values and variable importance. The most impactful feature of the prediction was the maximal value of VE during the test, up to 85% of age-predicted HR_max_. Additionally, subjects’ weight and sex influenced the prediction results, with higher VO_2peak_ observed in lighter individuals and males compared to females. The importance of weight as a predictor for VO_2peak_ in the presented study was probably due to the utilization of peak oxygen consumption in relation to mass and expressed in ml/min/kg, which is in line with the results presented by Loftin et al. [62]. There are also multiple studies presenting the difference in VO_2peak_ between male and female [63, 64], thus the influence of the patients sex on the prediction seem to be natural. Notably, there was no significant difference between the metrics obtained for both sexes, indicating the robustness of the models in this regard. Patients age was not among the most influential features, however a higher patients age tend to result in the lower value of the predicted VO_2peak_, which seem to be in line with the results of other studies [64, 65]. ML algorithms offer the advantage of processing and analyzing vast amounts of data at incredible speed, enabling them to identify complex patterns and relationships that may not be captured by humans. Thus, ML allows for the extraction of information on the influence of the almost unlimited number of features from big datasets on the VO_2peak_ values, which would be impossible just by human-based analysis. Notably, 13 out of the 20 features with the highest Shapley values and 10 out of the 15 features with the highest variable importance score were related to respiratory signals. Those findings seem to be in line with results presented in other studies, where the importance of respiratory signals in the context of oxygen consumption was presented [31, 66, 67]. The presented configuration offers the benefit of avoiding monitoring O_2_ consumption and CO_2_ production through laboratory device, instead allowing for the application of less sophisticated respiratory monitoring techniques, such as IP. Simultaneous acquisition of both ECG and IP can be performed using e.g., Pneumonitor device, which was recently developed and designed for research in the fields of physiology and sports medicine [12, 13, 68]. Thus, all the cardiorespiratory features under current study could be obtained using Pneumonitor without any additional equipment. However, it is important that the presented results are based on the CPET performed on a treadmill and machine learning models training in this study should not be used on a data from tests performed using other exercise modalities, as it influences the cardiorespiratory parameters [69]. Moreover, the profile of the study population in terms of age and fitness level of subjects should be considered if applying the obtained models, as it has an influence on the VO_2peak_ values [70].

There are several limitations of the study. First of all, the raw ECG/RR-intervals signals and raw respiratory curves were unavailable, and thus more sophisticated parameters especially from information and causal domains, which could provide additional insights into the predictive models could have not been calculated. Moreover, the sample size in this study was limited, as only 369 recordings from the initial database of 992 CPET recordings were used for analysis after applying exclusion criteria based on outlier detection methods and visual inspection of the signals. Furthermore, the dataset was imbalanced in terms of patients’ sex as there were 275 men and 52 women. A larger and more balanced dataset could prove beneficial for ML model training. There was also lack of information about the amount of sport activity undertaken by the participants, which might introduce inconsistency in the study population. Moreover, the equation used for determination of age-predicted HR_max_ might be also treated as a limitation as there exist equations with smaller errors [71]. However, the equation used in this study is the most popularity and characterized by simplicity of application. Additionally, one approach of age-predicted HR_max_ calculation and one threshold of HR_max_ were introduced. Some of these limitations could be overcome by the usage of the Pneumonitor device, which allows for the simultaneous acquisition of raw ECG and IP signals [68]. Thus, the pulmonary activity (including RespRate and VE) can be monitored without the usage of sophisticated apparatus for gas analysis and tight-fitting masks may stress some groups of patients (e.g., children). Future studies may explore the optimal percentage of HR_max_ and different approaches of estimation of age-predicted HR_max_. It would be also valuable to study the influence of the subjects’ physical activity level on the prediction accuracy as well as other than treadmill forms of cardiopulmonary exercise tests in order to determine the optimal settings for the prediction of VO_2peak_ for clinical practice. The utility of the described method may also depend on the reproducibility of the results, which need further testing. With high reproducibility, the method could be useful in clinical practice for e.g., tracking the changes of the CRF during training camps of athletes without performing full CPET.

This study expands the discussion on predicting cardiorespiratory fitness by highlighting the important role of submaximal testing and incorporating respiratory signals in the prediction process. The presented analysis indicates that the inclusion of respiratory parameters might improve the quality of the VO_2peak_ prediction in a group of athletic subjects aged between 18 and 40 years old when performing a submaximal test on a treadmill. The use of a submaximal test based on age-predicted HR_max_ and the utilization of cardiological and respiratory parameters that can be obtained without specialized CPET equipment is an advantage of the presented approach and facilitates its potential application in clinical practice.

## Supporting information

S1 Appendix List of feature names. (DOCX)
