# Supplementary material for: Prediction of peak oxygen consumption using cardiorespiratory parameters from warmup and submaximal stage of treadmill cardiopulmonary exercise test
Source: PLoS One. 2024 Jan 10;19(1):e0291706. doi: 10.1371/journal.pone.0291706 (PMC10781163; doi:10.1371/journal.pone.0291706)
Supplement: S1 Appendix — (DOCX) [file pone.0291706.s001.docx]

**S1 APPENDIX. List of feature names**

**VE_RUN_85_HRMAX_AGE_maximum** – maximal value of VE during the test up to 85% of age-predicted HR_max_

**Weight** – Subjects’ weight

**Sex** – Subjects’ sex

**VE_RUN_85_HRMAX_AGE_q75** – 75^th^ quantile of VE during the test up to 85% of age-predicted HR_max_

**RespRate_RUN_85_HRMAX_AGE_std** – maximal value of RespRate during the test up to 85% of age-predicted HR_max_

**HR_WARMUP_kurtosis** – kurtosis of the HR during the last 30 seconds of warmup

**VE_RUN_85_HRMAX_AGE_lr_alpha** – alpha coefficient from linear regression fitting of VE during the test up to 85% of age-predicted HR_max_

**VE_WARMUP_impuls_factor** – impulse factor from VE during the last 30 seconds of warmup

**ReapRate_RUN_85_HRMAX_AGE_q25** –75^th^ quantile of RespRate during the test up to 85% of age-predicted HR_max_

**HR_WARMUP_skewness** – skewness of the HR during last 30 seconds of warmup

**RespRate_WARMUP_lr_alpha** – alpha coefficient from linear regression fitting of RespRate during the last 30 seconds of warmup

**RespRate_WARMUP_skewness** – skewness of the RespRate during the last 30 seconds of warmup

**HR_RUN_85_HRMAX_AGE_q75** – 75^th^ quantile of HR during the test up to 85% of age-predicted HR_max_

**HR_WARMUP_q25** – 25^th^ quantile of HR during the last 30 seconds of warmup

**RespRate_RUN_85_HRMAX_AGE_lr_alpha** – alpha coefficient from linear regression fitting of RespRate during the test up to 85% of age-predicted HR_max_

**VE_RUN_RUN_85_HRMAX_AGE_mean** – mean value of VE during the test up to 85% of age-predicted HR_max_

**VE_RUN_85_HRMAX_AGE_q25** – 25^th^ quantile of VE during the test up to 85% of age-predicted HR_max_

**VE_WARMUP_kurtosis** – kurtosis of the VE during the last 30 seconds of warmup

**HR_RUN_85_HRMAX_AGE_max_min** – the difference between maximal and minimal HR value during the test up to 85% of age-predicted HR_max_

**HR_WARMUP_median** – median value of the HR during the last 30 seconds of warmup
